# Loss of Spike N370 glycosylation as an important evolutionary event for the enhanced infectivity of SARS-CoV-2

**DOI:** 10.1038/s41422-021-00600-y

**Published:** 2022-01-12

**Authors:** Shuyuan Zhang, Qingtai Liang, Xinheng He, Chongchong Zhao, Wenlin Ren, Ziqing Yang, Ziyi Wang, Qiang Ding, Haiteng Deng, Tong Wang, Linqi Zhang, Xinquan Wang

**Affiliations:** 1 The Ministry of Education Key Laboratory of Protein Science, Beijing Advanced Innovation Center for Structural Biology, Beijing Frontier Research Center for Biological Structure, School of Life Sciences, Tsinghua University, Beijing, China; 2 Comprehensive AIDS Research Center and Beijing Advanced Innovation Center for Structural Biology, School of Medicine, Tsinghua University, Beijing, China; 3 NexVac Research Center, Tsinghua University, Beijing, China; 4 Microsoft Research Asia, Beijing, China; 5 Protein Chemistry and Proteomics Facility Technology Center for Protein Research, Tsinghua University, Beijing, China; 6 Center for Infectious Disease Research and Beijing Advanced Innovation Center for Structural Biology, School of Medicine, Tsinghua University, Beijing, China

**Keywords:** Cryoelectron microscopy, Immunology

Dear Editor,

SARS-CoV-2 belongs to the *Sarbecovirus* subgenus of betacoronaviruses and other members in this subgenus include SARS-CoV and coronaviruses mainly found in bats^1^. It is generally believed that like SARS-CoV and MERS-CoV, SARS-CoV-2 has a natural origin and was selected either in animal hosts before zoonotic transfer, or in humans following zoonotic transfer^2^. However, the molecular determinants for host expansion of SARS-CoV-2 from animal reservoirs to humans remain largely unclear. The Spike (S) glycoprotein of coronaviruses mediates viral entry by binding host receptor and fusing viral and cellular membranes^3^. SARS-CoV-2 S glycoprotein contains 22 N-glycosylation sites and 17 O-glycosylation sites^4^. Cryo-EM structures of the bat RaTG13 and pangolin PCoV_GX coronavirus S trimers showed that, besides protein–protein interactions, one RBD is also contacted by N-glycans linked to three asparagine residues of the neighboring S protomer (N165 and N234 in the NTD and N370 in the RBD) (Supplementary information, Fig. S1a)^5^. However, the N370 glycosylation is lost in the SARS-CoV-2 S glycoprotein due to the threonine-to-alanine mutation at position 372 (Supplementary information, Fig. S1a). In contrast, the typical -NST- glycosylation motif is highly conserved among eight SARS-CoV-2-related *Sarbecovirus* members including SARS-CoV and coronaviruses from bat and pangolin (Supplementary information, Fig. S1b).

Here, we constructed wild-type (WT) SARS-CoV-2 pseudovirus and its mutant bearing the A372T mutation to restore the N370 glycosylation. The A372T mutant pseudovirus displayed about a 50-fold decrease in viral entry into HeLa cells expressing human ACE2 (hACE2) compared to WT (Fig. 1a). In parallel, we also constructed the RaTG13 and PCoV_GX pseudoviruses carrying respective T372A and T370A mutations to knock out the N370 and N368 glycosylation, respectively. The RaTG13 T372A and PCoV_GX T370A pseudoviruses respectively displayed 190-fold and 2-fold increases in their infectivity (Fig. 1a). In addition, the A372T mutation also substantially decreased the entry of SARS-CoV-2 pseudovirus into HeLa cells ectopically expressing ACE2 from other mammals (Fig. 1b). For RaTG13 T372A pseudovirus, HeLa cells expressing cat, pig, dog or mouse ACE2 were more easily infected compared to the WT pseudovirus (Fig. 1b). We also tested the infection of pseudoviruses into HeLa-hACE2 cells that were reported to endogenously express TMPRSS2 in the presence of TMPRSS2 inhibitor camostat mesylate^6^. As expected, the presence of camostat mesylate decreased the infection of all tested SARS-CoV-2, RaTG13 and PCoV_GX WT and mutant pseudoviruses (Supplementary information, Fig. S2). However, the inhibition of TMPRSS2 did not change the trend of decreased (SARS-CoV-2 A372T) or increased (RaTG13 T372A and PCoV_GX T370A) infection of the mutated pseudoviurses compared to their respective WT (Supplementary information, Fig. S2). Taken together, these results indicated that the loss of N370 glycosylation in SARS-CoV-2 is critical for its infectivity mediated by ACE2 of human and animal origin.

**Fig. 1:**
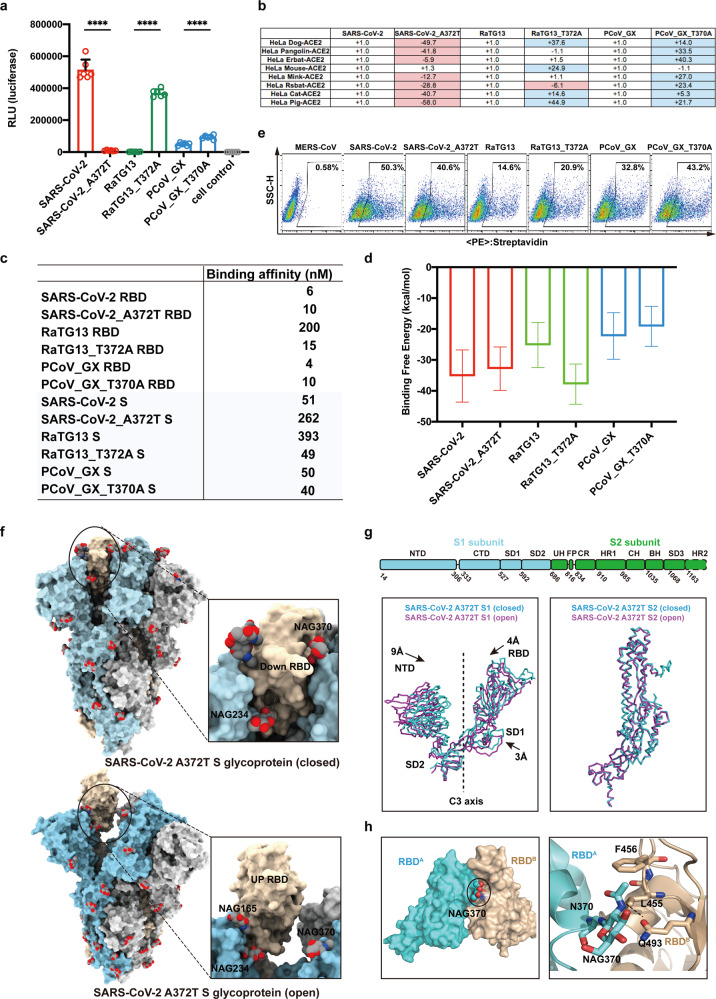
The effects of N370-linked glycans on the infectivity, binding to hACE2 and conformational state of the S glycoprotein. **a** Entry of the SARS-CoV-2, RaTG13 and PCoV_GX pseudoviruses and their mutants (SARS-CoV-2 A372T, RaTG13 T372A and PCoV_GX T370A) into HeLa cells expressing hACE2. Data represent the means ± SD of six replicates. ****P < 0.0001 (two-tailed unpaired *t*-test). **b** Entry efficiency of the SARS-CoV-2, RaTG13 and PCoV_GX pseudoviruses WT and mutants into HeLa cells expressing ACE2 from diverse host species. The values show the fold changes in luciferase activity for SARS-CoV-2 A372T, RaTG13 T372A and PCoV_GX T370A pseudoviruses compared to their respective WT. The symbol “+” indicates an increase in entry efficiency, while “–” indicates a decrease. Red highlights indicate at least threefold decrease in efficiency; blue indicates at least threefold increase in efficiency; white indicates changes no greater than threefold. Results were calculated from three independent experiments. **c** Binding affinities of the SARS-CoV-2, RaTG13, PCoV_GX RBDs, S trimers and their mutants with hACE2 measured by SPR method. **d** Binding free energies of the SARS-CoV-2, RaTG13 and PCoV_GX RBDs and their mutants with hACE2 estimated by MM/GBSA after MD simulation. **e** Binding to cell surface expressing hACE2 by the soluble S trimers of the SARS-CoV-2, RaTG13, PCoV_GX by FACS. The gated cell percentages are shown and the MERS-CoV S glycoprotein is used as negative control. **f** Overall structures of the SARS-CoV-2 A372T S glycoprotein with N-linked glycans. Top panel shows the closed S trimer with all three RBDs in the “down” conformation. Bottom panel shows the open S trimer with one “up” RBD and two “down” RBDs. Enlarged panels show interface surface between one RBD and NAG165, NAG234, NAG370 from the neighboring S monomer. The N-linked glycans are shown as spheres. **g** Schematic representation of the SARS-CoV-2 S glycoprotein structural domains (top panel) and comparisons of the S1 subunits (left panel) and the S2 subunits (right panel) containing “down” RBD of closed and open SARS-CoV-2 A372T S glycoproteins. The subunits of closed and open SARS-CoV-2 A372T S glycoproteins are colored in cyan and magenta, respectively. The shift distances of the NTD, RBD and SD1 towards the C3 axis are labeled. The C3 axis is the three-fold symmetry axis in the closed S trimer. **h** Location of NAG370 and interactions between NAG370 with residues L455, F456 and Q493 of the neighboring RBD. The residues are shown as sticks and the hydrogen bond between NAG370 and Q493 is shown as dotted line.

We expressed and purified WT and mutated RBD and S proteins of SARS-CoV-2, RaTG13 and PCoV_GX WT from HEK293F cells. The absence or presence of N370 glycosylation in the S proteins were confirmed by mass spectrometry (Supplementary information, Fig. S3). The binding affinities of WT and mutant RBD proteins to hACE2 were detected by surface plasmon resonance (SPR) method (Fig. 1c; Supplementary information, Fig. S4). The SARS-CoV-2 A372T mutation had minimal effect on the binding to ACE2, evidenced by WT and A372T RBDs displaying 6 nM and 10 nM affinities, respectively. Similarly, the T370A mutation did not result in significant alteration in ACE2 binding by PCoV_GX RBD. WT and mutated RBDs bound to ACE2 with affinities of 4 nM and 10 nM, respectively. In contrast, the T372A mutation resulted in much tighter ACE2 binding by RaTG13 T372A RBD than WT (15 nM vs 200 nM, 13-fold increase). In addition, we utilized Molecular Dynamic (MD) simulation and Molecular Mechanics Generalized Born Surface Area (MM/GBSA) method to estimate the binding free energies between various RBD proteins and ACE2 (Fig. 1d). The binding free energies of ACE2 to SARS-CoV-2 WT and A372T RBDs were –35.19 ± 8.43 and –32.84 ± 7.01 kcal/mol, respectively. PCoV_GX WT and T370A RBDs respectively exhibited binding free energies of –22.26 ± 7.53 and –19.12 ± 6.45 kcal/mol. However, the binding free energy of ACE2 to RaTG13 T372A RBD was markedly lower (–37.84 ± 6.51 kcal/mol) than to WT RBD (–25.20 ± 7.30 kcal/mol). These results showed that N370 glycosylation strongly influenced the receptor-binding ability of RaTG13 RBD, whereas the effects on SARS-CoV-2 and PCoV_GX RBDs were mild.

The effects of mutations on the receptor binding ability of the RaTG13 and PCoV_GX S trimers and their respective RBDs are similar (Fig. 1c and Supplementary information, Fig. S4). The T372A mutation resulted in an 8-fold increase in ACE2 binding by RaTG13 S mutated trimer compared to WT (49 nM vs 393 nM). The PCoV_GX S WT and T370A mutant, however, exhibited the similar level of binding to ACE2 (50 nM vs 40 nM). The SARS-CoV-2 S protein bearing A372T mutation displayed a 5-fold decrease in ACE2 binding compared to WT (262 nM vs 51 nM), whereas the same mutation had nearly no effect on the binding of RBD to ACE2, suggesting that A372T mutation had more impact on ACE2 binding in the context of trimeric S protein than RBD. We further performed the fluorescence-activated cell sorting (FACS) by staining the HEK293T cells expressing hACE2 with soluble S glycoprotein. The FACS results also showed that the staining by the SARS-CoV-2 S A372T mutant was reduced compared to WT, whereas the staining by the RaTG13 T372A and PCoV_GX T370A was increased (Fig. 1e).

The cryo-EM structures of the RaTG13, PCoV_GX and PCoV_GD S trimers were all only solved in the closed state, in which the three RBDs are in the “down” conformation and the ACE2-binding sites are buried^5, 7^. In addition to the closed state, the SARS-CoV-2 S trimer always exhibits another open state, in which one RBD is in the “up” conformation to expose the ACE2-binding site^8^. We collected the single-particle cryo-EM data for both A372T (2149 images) and WT (661 images) S trimers. The 3D-classification showed that the percentages of SARS-CoV-2 S trimers in the closed and open states were 60% (117,867 particles) and 37% (72,563 particles), respectively. After introducing the A372T mutation, the percentage of the closed S trimers was increased to 84% (348,420 particles), and the percentage of the open S trimers was reduced to 14% (55,181 particles). The loss of N370 glycosylation therefore facilitates for the SARS-CoV-2 S trimers to be in the open state essential for ACE2 binding.

Two atomic models of the SARS-CoV-2 S A372T trimers were built with one in the closed state at 3.1 Å resolution and the other one in the open state at 3.9 Å resolution (Fig. 1f; Supplementary information, Figs. S5, S6 and Table S1). In the closed S trimer, all three RBDs are in the “down” conformation, and the open trimer has one RBD in the “up” conformation (Fig. 1f). Structural alignment of the S monomers containing “down” RBD from two different S trimers revealed the root mean square deviation (r.m.s.d.) of 2.50 Å for all aligned Cα atoms. The S2 subunit is more structurally conserved with an r.m.s.d. of 0.48 Å than the S1 subunit with an r.m.s.d. of 3.86 Å. When the S1 subunits containing “down” RBD from two different S trimers were superimposed, the NTD, RBD and SD1 in the closed S trimer all moved inward to the three-fold axis compared to the open S trimer, and the shift distances of the NTD, RBD and SD1 are around 9 Å, 4 Å and 3 Å, respectively (Fig. 1g). Therefore, the closed A372T S trimer is more compact than the open one. The NAG370 locates at the RBD-RBD interface in the closed S trimer, and interactions were observed between NAG370 and residues L455, F456 and Q493 of the neighboring RBD (Fig. 1f and h). Specifically, hydrogen-bonding interactions occurred between NAG370 and RBD Q493 (Fig. 1h). In the open trimer, NAG370 does not contact the RBD in the “up” conformation (Fig. 1f).

In this study, we revealed that by removing the N370 glycan through T372A mutation during evolution, SARS-CoV-2 adopted more desirable open state, thereby facilitating more efficient binding to ACE2 and higher capacity for infection. Through comparisons with the closely related bat RaTG13 and pangolin PCoV_GX coronaviruses, we further supported our previous proposal that tight RBD–ACE2 binding and efficient RBD “down” to “up” conformational transition are both required for SARS-CoV-2 to efficiently infect and transmit among humans^5^. While this paper was under preparation for submission, Kang et al. reported a scanning over 182,000 SARS-CoV-2 genomes for selective sweep signatures and the identification of the same T372A change^9^. They showed that the A372T reversion mutation resulted in decreased viral replication in human lung cells and proved a decreased binding of the A372T mutant S glycoprotein to ACE2 in an ELISA-based binding assay^9^. The presence of the N370-linked glycans favoring the SARS-CoV-2 S glycoprotein to be in the closed state has also been proposed by Harbison et al.^10^. Our results, together with these two recent studies, indicated the loss of N370 glycosylation in the S glycoprotein as an important evolutionary event for the enhanced infectivity of SARS-CoV-2.

## Supplementary information

Supplementary Information

## Data Availability

The atomic coordinates of SARS-CoV-2 A372T S glycoprotein have been deposited in the Worldwide Protein Data Bank with the accession codes 7FCE (closed state) and 7FCD (open state), respectively; the corresponding maps have been deposited in the Electron Microscopy Data Bank with the accession codes EMD-31525 and EMD-31524, respectively.
