# Supplementary material for: Loss of Spike N370 glycosylation as an important evolutionary event for the enhanced infectivity of SARS-CoV-2
Source: Cell Res. 2022 Jan 12;32(3):315–8. doi: 10.1038/s41422-021-00600-y (PMC8752327; doi:10.1038/s41422-021-00600-y)
Supplement: Supplementary file 1 — Supplementary Information [file 41422_2021_600_MOESM1_ESM.pdf]

Supplementary information

Materials and Methods

Production of pseudoviruses and infection assay

The cDNAs encoding the S glycoproteins of SARS-CoV-2 (GenBank: YP_009724390.1), RaTG13 (GenBank: QHR63300.2) and PCoV_GX (GenBank: QIA48614.1) were used for pseudovirus production. The SARS-CoV-2_A372T, RaTG13_T372A and PCoV_GX_T370A S mutants were generated by site-specific PCR mutagenesis. All the S sequences were introduced into the pcDNA3.1 vector. The pseudoviruses were generated by co-transfecting HEK-293T cells (ATCC) with human immunodeficiency virus backbones expressing firefly luciferase (pNL4-3-R-E-79 luciferase) and pcDNA3.1 vector encoding either wild type (WT) or mutated S glycoprotein. Viral supernatant was collected 60 h later, centrifuged to remove cell lysis, and stored at -80°C until use.

The pseudoviruses of SARS-CoV-2, RaTG13, PCoV_GX and their mutants were normalized by p24 ELISA kit (KEY-BIO). Then, HeLa-hACE2 cells were added to 96 well plates and mixed with 50 ul of pseudovirus. For analysis of the TMPRSS2 inhibition experiment, HeLa-hACE2 cells were pretreated with Camostat mesylate (100 μM) for 3 h. The luciferase activities were analyzed 60 h after infection using Bright-Glo 92 Luciferase Assay Vector System (Promega Bioscience). Uninfected HeLa-hACE2 cells were used as controls. Each group contains 6 replicates.

HeLa cell lines expressing ACE2 from other mammals

HeLa cells expressing ACE2 orthologs were kindly provided by Dr. Qiang Ding at Tsinghua University School of Medicine. The cDNAs encoding ACE2 orthologs were synthesized by GenScript and cloned into pLVX-IRES-zsGreen1 vectors (Catalog No. 632187, Clontech Laboratories, Inc) with a C-terminal FLAG tag. VSV-G pseudotyped lentiviruses expressing ACE2 orthologs were produced and used to generate HeLa-ACE2 cells as previously described[1]. For studying entry efficiency of the SARS-CoV-2, RaTG13 and PCoV_GX pseudoviruses and their mutants, 50 μl of each pseudovirus was added to each well of the 96 well plate containing HeLa-ACE2

cells expressing different ACE2 orthologs, and the luciferase activities were analyzed 60 h after infection using Bright-Glo Luciferase Assay Vector System (Promega Bioscience). Absolute and fold changes between mutant and WT were used to estimate the influence of entry efficiency.

Construct design for protein expression

The SARS-CoV-2 S ectodomain (1-1208), RaTG13 S ectodomain (1-1209) and PCoV_GX S ectodomain (1-1205) were cloned into the pCAG vector separately. All the constructs included a C-terminal foldon tag for trimerization, a Strep tag for purification and '2P' mutations (K986P and V987P for SARS-CoV-2; K982P and V983P for RaTG13; K980P and V981P for PCoV_GX). The SARS-CoV-2 RBD (333-527), PCoV_GX RBD (331-524) and RaTG13 RBD (333-526) were inserted into the pCAG vector with an N-terminal CD4 signal peptide for secretion and a C-terminal 6×His tag for purification. The SARS-CoV-2_A372T, RaTG13_T372A and PCoV_GX_T370A S and RBD mutants were generated by site-specific PCR mutagenesis and cloned into the pCAG vector. The hACE2 extracellular domain (19-615) were inserted into the pFastBac-Dual vector, with an N-terminal gp67 signal peptide for secretion and a C-terminal 6×His tag for purification.

Protein expression and purification

The SARS-CoV-2, RaTG13 and PCoV_GX WT and mutated S and RBD proteins were expressed in FreeStyle 293-F cells (Invitrogen) cultured in suspension in 5% CO2 atmosphere, at 37°C with shaking at 120 rpm. Cell cultures were transiently transfected with 1 mg of plasmid per liter of culture at a density of 2×10^6/ml using polyethylenimine (PEIs) (Sigma), and the supernatants were collected 96 hours later. The hACE2 were produced in Hi5 insect cells using the Bac-to-Bac baculovirus system (Invitrogen) as previously described[2].

The supernatants containing SARS-CoV-2, RaTG13, PCoV_GX S glycoproteins and their mutants were concentrated and exchanged to HBS buffer (10 mM HEPES, pH 7.2, 150 mM NaCl), purified by StrepTactin beads (IBA) and then purified by gel-filtration chromatography using a Superose 6 column (GE Healthcare) pre-equilibrated with HBS buffer. The cell medium containing hACE2, SARS-CoV-2

RBD, RaTG13 RBD, PCoV_GX RBD and their mutants were concentrated and exchanged to HBS buffer, purified by Ni-NTA resin (GE Healthcare), and followed by size-exclusion chromatography using a Superdex 200 column (GE Healthcare) pre-equilibrated with HBS buffer.

Cryo-EM sample preparation and data collection.

Aliquots of SARS-CoV-2_A372T S ectodomain (4 μl, 0.8 mg/ml, in HBS buffer) were applied to glow-discharged holey carbon grids (Quantifoil grid, Cu 300 mesh, R1.2/1.3). The grids were then blotted and plunge-frozen into liquid ethane using Vitrobot Mark IV (Thermo Fisher Scientific).

Images for complex were recorded using FEI Titan Krios microscope (Thermo Fisher Scientific) operating at 300 kV with a Gatan K3 Summit direct electron detector (Gatan Inc.) at Tsinghua University. The automated software (AutoEMation2)[3] was used to collect 2149 movies for complex in super-resolution mode at a nominal magnification of 64,000× and at a defocus range between -1.5 and -1.8 μm. Each movie has a total accumulate exposure of 50 e-/Å2 fractionated in 32 frames of 175 ms exposure. The final image was binned 2-fold to a pixel size of 1.0979 Å. Data collection statistics are summarized in Supplementary Table 1.

Cryo-EM data processing

Motion Correction (MotionCor2 v.1.2.6)[4], CTF-estimation (GCTF v.1.18)[5] and non-templated particle picking (Gautomatch v.0.56, http://www.mrc-lmb.cam.ac.uk/kzhang/) were automatically executed by TsingTitan.py program. Sequential data processing was carried out on RELION-3.1[6]. After 2D classification, the best selected 928,349 particles were applied for initial model and 3D classification. A subset of 348,420 particle images from state 1 (closed S glycoprotein) and 55,181 particle images from state 2 (open S glycoprotein) were further subject to 3D auto-refine and post-processing. The final resolution for state 1 and state 2 are 3.1 Å and 3.9 Å, respectively. The resolution was estimated with the gold-standard Fourier shell correlation 0.143 criterion. Refer to Fig S1 and Supplementary Table 1 for details of data collection and processing.

Model building and refinement

The initial model of SARS-CoV-2_A372T S ectodomain was generated using the models (PDB 7VXX and 7VYB) and fit into the map using UCSF Chimera v.1.15[7]. Manual model rebuilding was carried out using Coot v.0.9.2[8] and refined with PHENIX v.1.18.2[9] real-space refinement. The quality of the final model was analyzed with PHENIX v.1.18.2 and the glycan validation used Privateer[10]. The validation statistics of the structural models are summarized in Supplementary Table 1. All structural figures were generated using PyMOL 2.0[11] and ChimeraX-1.1.1[12].

Mass spectrometry analysis of glycopeptides

The 10 ug S glycoproteins were separated by SDS-PAGE. The gel bands of interest were excised from the gel, reduced with 5 mM of DTT and alkylated with 11 mM iodoacetamide which was followed by in-gel digestion with sequencing grade modified trypsin in 50 mM ammonium bicarbonate at 37℃ overnight. The next day, the sample was quenched by adding 10% TFA to adjust PH to below 2. Then the sample was digest with sequencing grade modified trypsin in 50 mM ammonium bicarbonate at 37℃overnight. The peptides were extracted twice with 0.1% trifluoroacetic acid in 50% acetonitrile aqueous solution for 1h and then dried in a speedVac. Peptides were redissolved in 25μl 0.1% trifluoroacetic acid and 6μl of extracted peptides were analyzed by Q Exactive HF-X mass spectrometer.

For LC-MS/MS analysis, the peptides were separated by a 120 min gradient elution at a flow rate 0.30 µl/min with a Thermo-Dionex Ultimate 3000 HPLC system, which was directly interfaced with an Q Exactive HF-X mass spectrometer (Thermo Fisher Scientific, Bremen, Germany). The analytical column was a home-made fused silica capillary column (75 µm ID, 150 mm length; Upchurch, Oak Harbor, WA) packed with C-18 resin (300 Å, 5 µm, Varian, Lexington, MA). Mobile phase A consisted of 0.1% formic acid, and mobile phase B consisted of 100% acetonitrile and 0.1% formic acid. The Q Exactive HF-X mass spectrometer was operated in the data-dependent acquisition mode using Xcalibur 4.1.31.9 software and there is a single full-scan mass spectrum in the Orbitrap (300–1800 m/z, 60,000 resolution) followed by by top-speed MS/MS scans in the Orbitrap.Fragmentation was performed with a normalized collision energy of 27.

Glycopeptide fragmentation data were extracted from the raw file using Byonic™ (Version 2.11.0). The MS data was searched using the Protein Metrics 309 N-glycan library. The search criteria were as follows: Full trypsin specific was required; carbamidomethylation (C) were set as the fixed modifications; the oxidation (M) was set as the variable modification; precursor ion mass tolerances were set at 20 ppm for all MS acquired in an orbitrap mass analyzer; and the fragment ion mass tolerance was set at 0.02 Da for all MS2 spectra acquired.

Surface plasmon resonance experiments

Running buffer composed of 10 mM HEPES pH 7.2, 150 mM NaCl and 0.05% (v/v) Tween-20 was used during the analysis and all proteins were exchanged to the same buffer. The purified hACE2 was covalently immobilized to a CM5 sensor chip (GE Healthcare) in 10 mM sodium acetate buffer (pH 4.5) using Biacore T200 (GE Healthcare). The blank channel of the chip was used as the negative control. Serial dilutions of SARS-CoV-2, RaTG13, PCoV_GX WT and mutated S and RBD proteins were flowed through the chip sequentially. The resulting data were analyzed using Biacore T200 Evaluation Software 3.1 (GE Healthcare) by fitting to a 1:1 binding model.

Construction of the MD simulation systems

The SARS-CoV-2 RBD-hACE2 complex structure (PDB ID: 6M0J) was used as initial model for simulation. And the RBD structures of RaTG13 and PCoV_GX were obtained from the RaTG13 S structure (PDB ID: 7CN8) and PCoV_GX S structure (PDB ID: 7CN4). The complex structures of RaTG13 RBD-hACE2 and PCoV_GX RBD-hACE2 were generated from SWISS-MODEL[13] using SARS-CoV-2 RBD-hACE2 complex structure (PDB ID: 6M0J) as template. All mutant systems were constructed based on the corresponding WT systems. The glycosylation was accomplished by the web server http://glycam.org.

Amber ff14SB force field was employed to model proteins[14] and GLYCAM06j force field was applied for glycosylation sites[15]. Then, a 20 Å TIP3P water box around the RBD-ACE2 complex was added to each simulation system and Na^+ counterions were added to the water boxes for neutralization[16]. At last, 0.15 mol/L

NaCl was added to the solvent to maintain our systems under physiological conditions.

Simulation runs

For each system, two rounds of energy minimization were firstly employed. In the initial minimization, the coordinates of solvents were optimized 5,000 steps with a restraint of 500 kcal/(mol*$Å^2$) on the RBD-ACE2 complex. Next, 10,000 steps of energy minimization were applied to the systems without any restraint. After minimization, systems were heated to 300K in 500 ps simulation under NVT condition and a restraint of 10 kcal/(mol*$Å^2$) on the complex structures. Then, two equilibration runs were carried out. In the first process, the coordinates of proteins were constrained by restraint of 10 kcal/(mol*$Å^2$), and the solvents were equilibrated for 1 ns. As a contrast, the restraint was removed in the second process and all atom coordinates encountered equilibration run. Finally, 100 ns MD simulations with a timestep of 2.0 fs were employed at 300 K temperature and 1 atm pressure, which were controlled by Langevin thermostat and isotropic position scaling, respectively. Long-range electrostatic interactions were treated by the Particle Mesh Ewald (PME) method[17], whereas a cutoff value of 10 Å was applied to calculate other electrostatic and van der Waals interactions. The SHAKE algorithm was also used to constrain all covalent bonds with hydrogen atoms[18]. The MD simulations was executed by AMBER20 pmemd.cuda program[19].

Estimation of the binding free energy

Molecular Mechanics Generalized Born Surface Area (MMGBSA) approach[20] was invoked to estimate the binding affinity of RBD towards ACE2. The total binding free energy, namely $\Delta G_{binding}$, were provided by equation (1)

$$\Delta G_{binding} = G_{complex} - (G_{ACE2} + G_{RBD}) \quad (1)$$

According to the second law of thermodynamics, $\Delta G_{binding}$ is decomposed into enthalpy (ΔH) and entropy ($-T\Delta S$) items. As shown in equation (2), the enthalpy part can be transferred to the sum of the energies of molecular mechanical (ΔE_{MM}) and the solvation free energy ($\Delta G_{solvation}$).

$$\Delta G_{binding} = \Delta H - T\Delta S = \Delta E_{MM} + \Delta G_{solvation} - T\Delta S \qquad (2)$$

Equation (3) represents that the energies of molecular mechanical include the internal energy (ΔE_{int}, including bond, angle and dihedral energies), van der Waals item (ΔE_{vdw}), and electrostatic item (ΔE_{ele}).

$$\Delta E_{MM} = \Delta E_{int} + \Delta E_{vdw} + \Delta E_{ele} \qquad (3)$$

In equation (4), $\Delta G_{solvation}$ is the sum of the polar part (ΔG_{polar}) and the non-polar part ($\Delta G_{nonpolar}$) items.

$$\Delta G_{solvation} = \Delta G_{polar} + \Delta G_{nonpolar} \qquad (4)$$

The Generalized Born^{OBC} model II was used to calculate the polar solvation energy with ionic strength of 0.15 mol/L[21]. The nonpolar item solvation energy was calculated based on the solvent accessible surface area (SASA) that was estimated by the Linear Combinations of Pairwise Overlaps (LCPO) model in equation (5).

$$\Delta G_{nonpolar} = \gamma SASA \qquad (5)$$

$\gamma = 0.005\ kcal \cdot mol^{-1} \cdot Å^{-2}$,

The script MMPBSA.py in Amber20 package was applied to accomplish the binding free energy calculations of the simulation systems[22].

Binding of S glycoproteins to cell surface expressed hACE2

HEK 293T cells were transfected with expression plasmid encoding full-length hACE2, and incubated at 37 °C for 24 h. Cells were digested from the plate with trypsin and distributed onto 96-well plates. Cells were washed thrice with 200 μL staining buffer (PBS with 1% heated-inactivated fetal bovine serum (FBS)) between each of the following steps. First, cells were stained with the recombinant strep-tagged S glycoprotein(10μg/ml) at 4 °C in 100 μl staining buffer. After 30 min, cells were washed and stained with PE-labeled streptavidin (Invitrogen) at 4 °C for 30 min. After sufficient washes, the cells were resuspended and analyzed with BD Calibur FACS cytometer (BD, USA) and FlowJo 10 software (FlowJo, USA). HEK 293T cells with mock transfection were stained as background control. MERS-CoV S glycoprotein were used as negative control.

References

1 Liu, Y. et al. Functional and genetic analysis of viral receptor ACE2 orthologs reveals a broad potential host range of SARS-CoV-2. Proc Natl Acad Sci U S A 118, doi:10.1073/pnas.2025373118 (2021).
2 Zhang, S. et al. Bat and pangolin coronavirus spike glycoprotein structures provide insights into SARS-CoV-2 evolution. Nat Commun 12, 1607, doi:10.1038/s41467-021-21767-3 (2021).
3 Lei, J. & Frank, J. Automated acquisition of cryo-electron micrographs for single particle reconstruction on an FEI Tecnai electron microscope. J Struct Biol 150, 69-80, doi:10.1016/j.jsb.2005.01.002 (2005).
4 Zheng, S. Q. et al. MotionCor2: anisotropic correction of beam-induced motion for improved cryo-electron microscopy. Nat Methods 14, 331-332, doi:10.1038/nmeth.4193 (2017).
5 Zhang, K. Gctf: Real-time CTF determination and correction. J Struct Biol 193, 1-12, doi:10.1016/j.jsb.2015.11.003 (2016).
6 Zivanov, J. et al. New tools for automated high-resolution cryo-EM structure determination in RELION-3. Elife 7, doi:10.7554/eLife.42166 (2018).
7 Pettersen, E. F. et al. UCSF Chimera--a visualization system for exploratory research and analysis. J Comput Chem 25, 1605-1612, doi:10.1002/jcc.20084 (2004).
8 Emsley, P. & Cowtan, K. Coot: model-building tools for molecular graphics. Acta Crystallogr D Biol Crystallogr 60, 2126-2132, doi:10.1107/S0907444904019158 (2004).
9 Adams, P. D. et al. PHENIX: a comprehensive Python-based system for macromolecular structure solution. Acta Crystallogr D Biol Crystallogr 66, 213-221, doi:10.1107/S0907444909052925 (2010).
10 Agirre, J. et al. Privateer: software for the conformational validation of carbohydrate structures. Nature Structural & Molecular Biology 22, 833.
11 Giacomo, J., Zhang, C., Giulia, P. M. & Alessandro, P. PyMod 2.0: improvements in protein sequence-structure analysis and homology modeling within PyMOL. Bioinformatics, 444 (2017).
12 Pettersen, E. F., Goddard, T. D., Huang, C. C., Meng, E. C. & Ferrin, T. E. UCSF ChimeraX: Structure Visualization for Researchers, Educators, and Developers. Protein Science 30(1), 70-82, doi:10.1002/pro.3943 (2020).
13 Waterhouse, A. et al. SWISS-MODEL: homology modelling of protein structures and complexes. Nucleic Acids Res 46, W296-W303, doi:10.1093/nar/gky427 (2018).
14 Maier, J. A. et al. ff14SB: Improving the Accuracy of Protein Side Chain and Backbone Parameters from ff99SB. J Chem Theory Comput 11, 3696-3713, doi:10.1021/acs.jctc.5b00255 (2015).
15 Kirschner, K. N. et al. GLYCAM06: a generalizable biomolecular force field. Carbohydrates. J Comput Chem 29, 622-655, doi:10.1002/jcc.20820 (2008).
16 Jorgensen, W. L., Chandrasekhar, J., Madura, J. D., Impey, R. W. & Klein, M. L. Comparison of simple potential functions for simulating liquid water. The Journal of Chemical Physics 79, 926-935, doi:10.1063/1.445869 (1983).

17 Darden et al. Particle mesh Ewald: An N·log(N) method for Ewald sums in large systems. Journal of Chemical Physics 98 (1993).

18 Ryckaert, J.-P., Ciccotti, G. & Berendsen, H. J. C. Numerical integration of the cartesian equations of motion of a system with constraints: molecular dynamics of n-alkanes. Journal of Computational Physics 23, 327-341, doi:10.1016/0021-9991(77)90098-5 (1977).

19 Salomon-Ferrer, R., Gotz, A. W., Poole, D., Le Grand, S. & Walker, R. C. Routine Microsecond Molecular Dynamics Simulations with AMBER on GPUs. 2. Explicit Solvent Particle Mesh Ewald. J Chem Theory Comput 9, 3878-3888, doi:10.1021/ct400314y (2013).

20 Genheden, S. & Ryde, U. The MM/PBSA and MM/GBSA methods to estimate ligand-binding affinities. Expert Opin Drug Discov 10, 449-461, doi:10.1517/17460441.2015.1032936 (2015).

21 Onufriev, A., Bashford, D. & Case, D. A. Exploring protein native states and large-scale conformational changes with a modified generalized born model. Proteins 55, 383-394, doi:10.1002/prot.20033 (2004).

22 Miller, B. R., 3rd et al. MMPBSA.py: An Efficient Program for End-State Free Energy Calculations. J Chem Theory Comput 8, 3314-3321, doi:10.1021/ct300418h (2012).

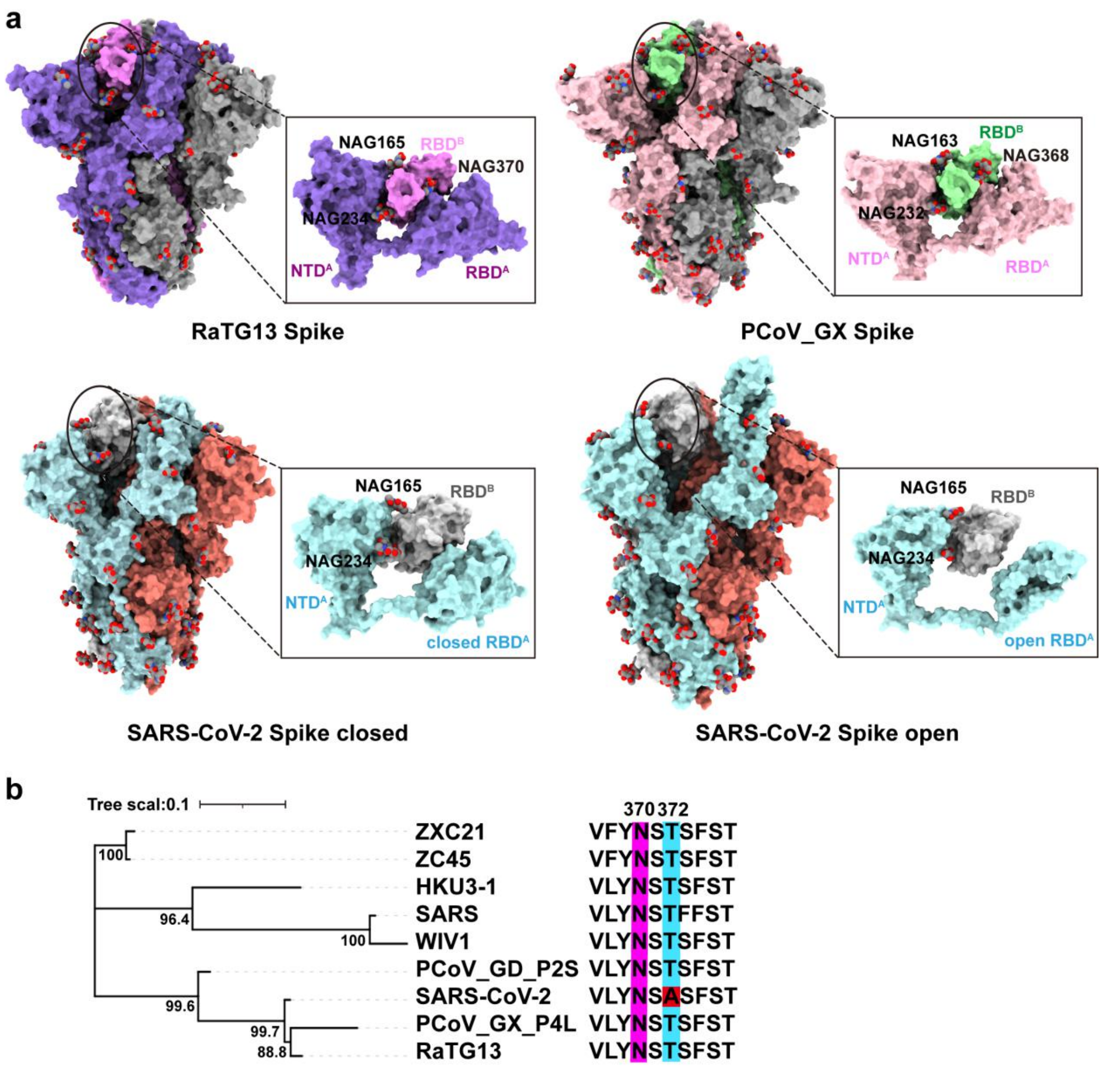

Supplementary information, Fig. S1 The unique loss of N370 glycosylation in the SARS-CoV-2 S glycoprotein due to the T372A mutation, which is not found in other related members in the *Sarbecovirus* subgenus. a Structures of the RaTG13, PCoV_GX and SARS-CoV-2 S glycoproteins. The RaTG13 S trimer (PDB ID:7CN4) and PCoV_GX S trimer (PDB ID:7CN8) are in the closed state, whereas the SARS-CoV-2 S trimer exhibits the closed state (PDB ID: 6VXX) and open state (PDB ID: 6VYB). Enlarged panels show the N-glycans around one RBD in the S glycoprotein trimer. **b** Phylogeny analysis of eight SARS-CoV-2 related members in the Sarbecovirus subgenus based on S using PhyML and amino acid sequence alignment around the N370 position. The numbers above the branches are percentage bootstrap values. The amino acid numbering 370 and 372 according to the SARS-CoV S sequence are indicated above the sequences.

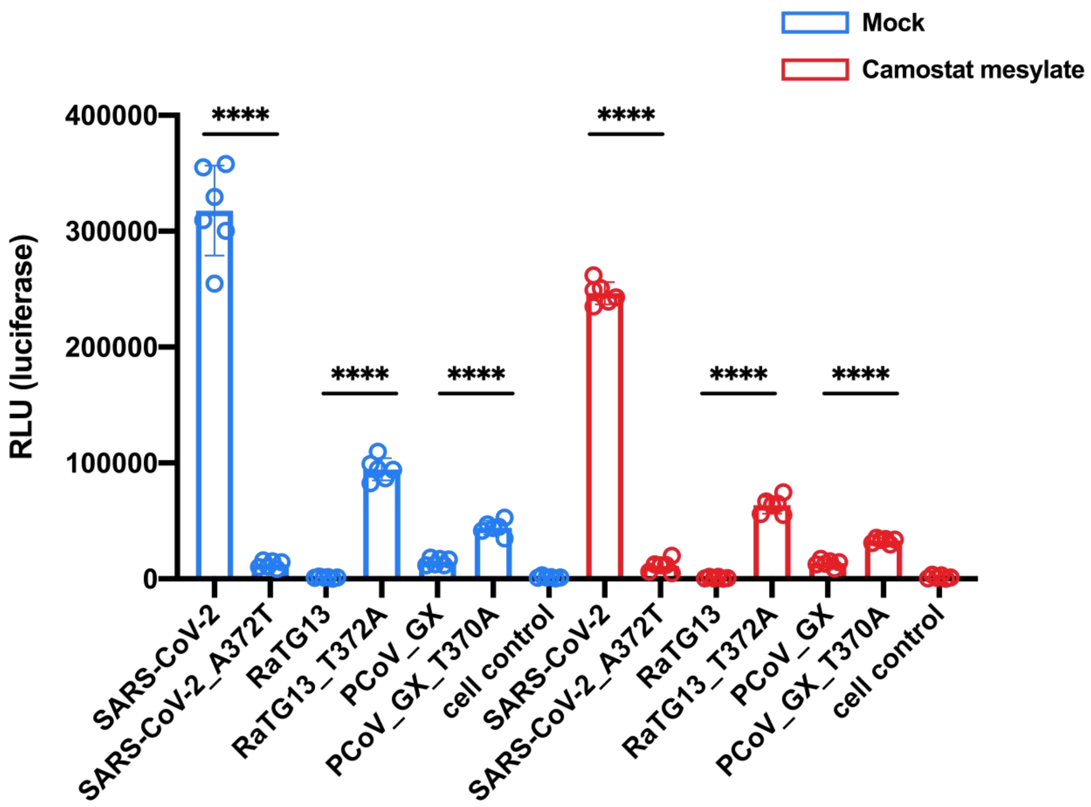

Supplementary information, Fig. S2 Entry of the SARS-CoV-2, RaTG13 and PCoV_GX pseudoviruses and their mutants bearing T372A/T370A mutations into HeLa-hACE2 cells in the presence of TMPRSS2 inhibitor camostat mesylate (100 µM). Data represent the mean ± SD of six replicates. ****$p < 0.0001$ (two-tailed unpaired t test).

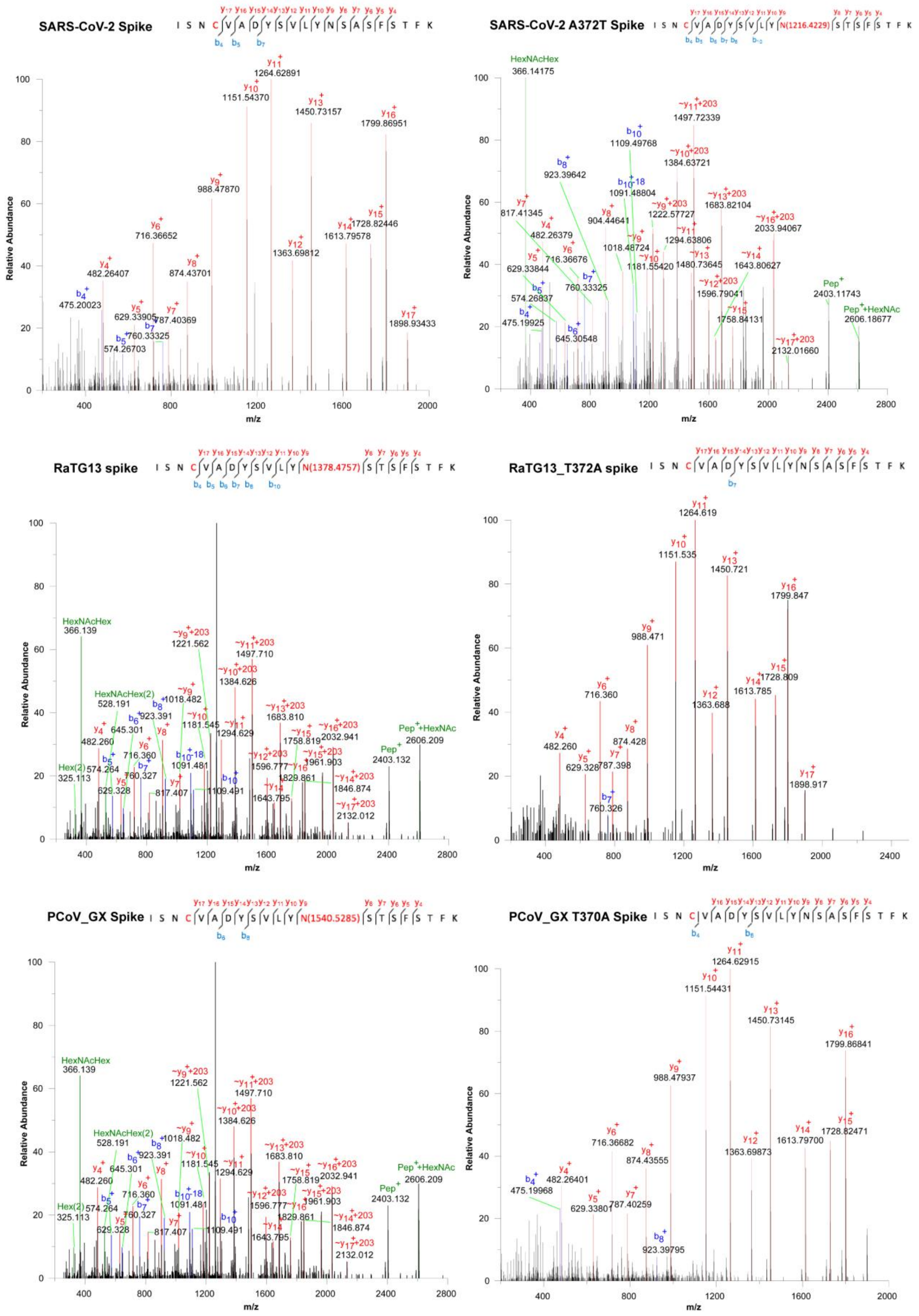

Supplementary information, Fig. S3 The MS/MS spectra of Higher-energy Collisional Dissociation (HCD) fragmented glycopeptides of the SARS-CoV-2, RaTG13, PCoV_GX S glycoproteins and their matants. Glycan oxonium ions at m/z 366 is HexNAcHex. MS/MS spectrum at m/z 2403 is peptide precursor ions

without glycans and m/z 2606 is peptide ions with HexNAc. None glycan fragments were identified in the SARS-CoV-2 S N370 position, RaTG13_T372A S N370 position and PCoV_GX_T370A S N368 position. The amino acid sequences are shown on the top of the panel.

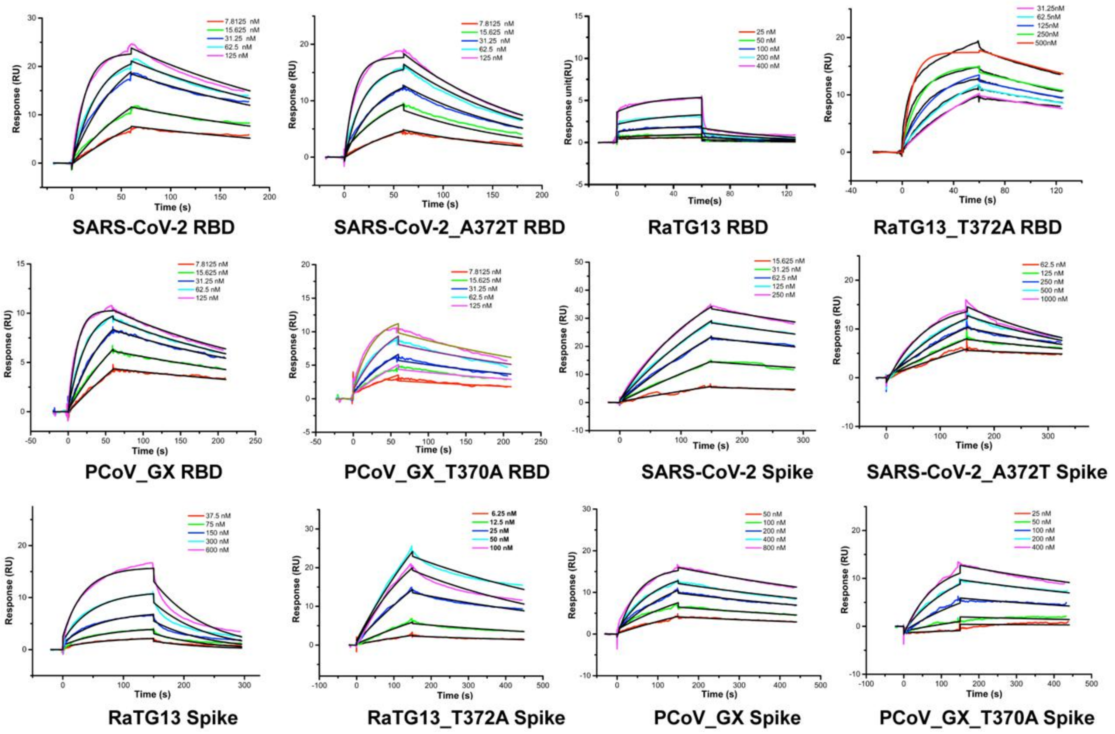

Supplementary information, Fig. S4 Surface plasmon resonance binding curves of RBDs and S glycoproteins to immobilized hACE2. Data are shown as different colored lines and the best fit of the data to a 1:1 binding model is shown in black.

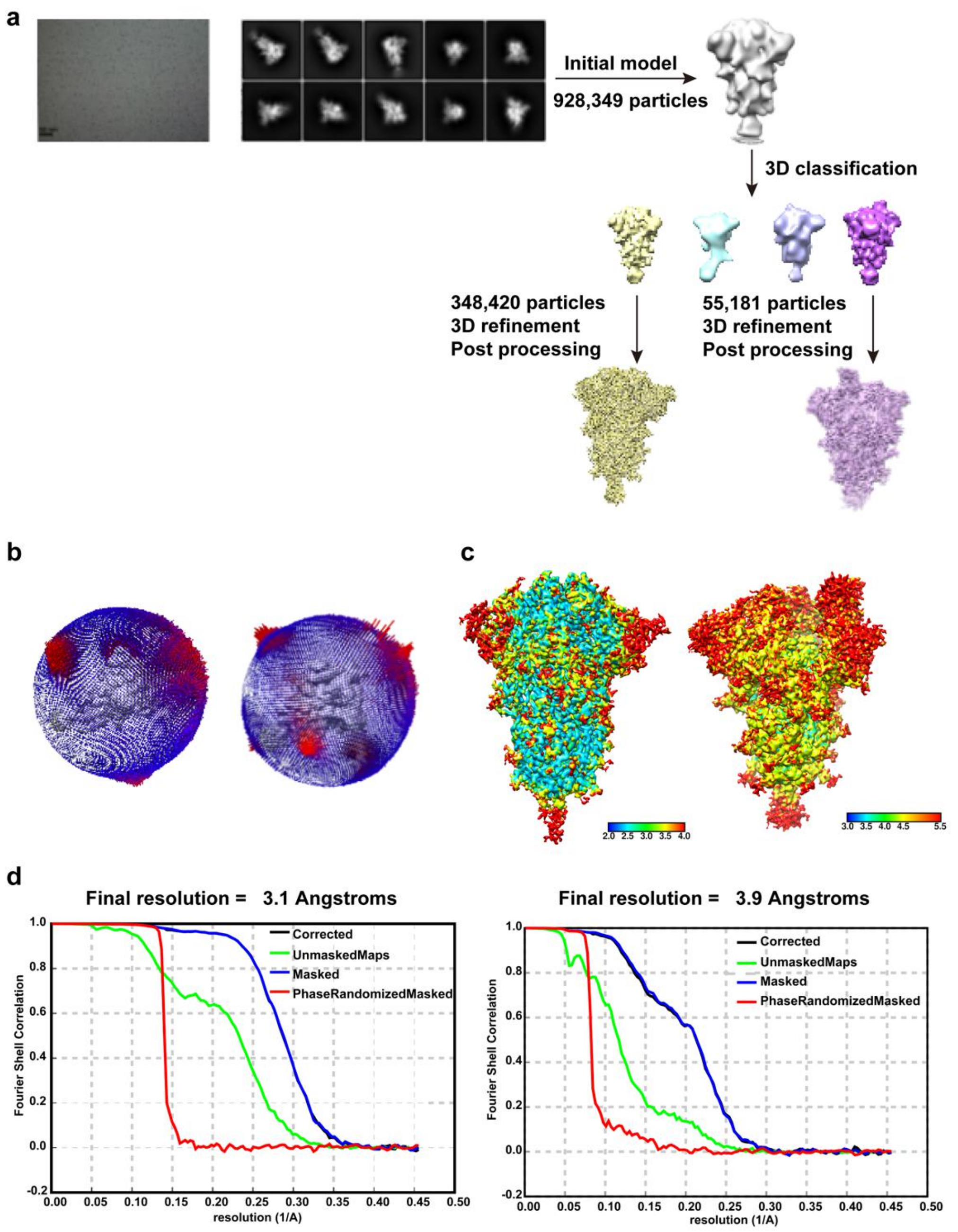

Supplementary information, Fig. S5 Cryo-EM data processing workflow and structure validations. a Processing workflow of the SARS-CoV-2 A372T S glycoprotein cryo-EM data. **b** Particle orientation distribution of the closed SARS-CoV-2 A372T S glycoprotein (left panel) and open SARS-CoV-2 A372T S glycoprotein (right panel). **c** Local resolution map of the closed SARS-CoV-2 A372T S glycoprotein trimer (left panel) and open SARS-CoV-2 A372T S glycoprotein trimer (right panel). The color scales indicate resolution. **d** FSC of the corrected, unmasked, masked and phase randomised for the cryo-EM reconstructions of the

density maps of the closed SARS-CoV-2 A372T S glycoprotein (left panel) and open SARS-CoV-2 A372T S glycoprotein (right panel). The resolution was estimated with the gold-standard Fourier shell correlation 0.143 criterion.

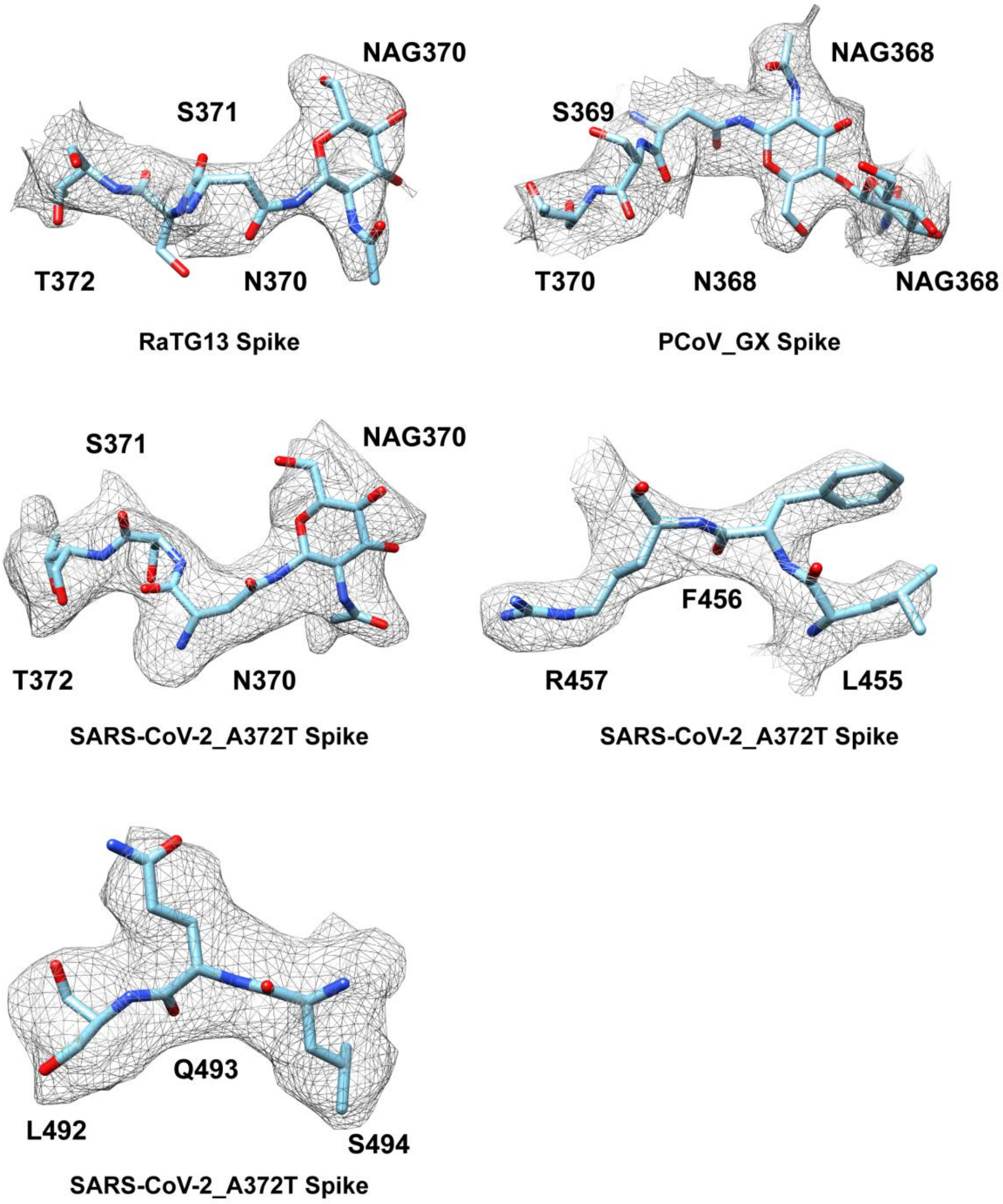

Supplementary information, Fig. S6 The representative density maps of NAG370 in the RaTG13 S, NAG368 in the PCoV_GX S, NAG370 and amino acid residues in the SARS-CoV-2 A372T S.

Supplementary information, Table S1 Cryo-EM data collection, refinement, and validation statistics

Data collection and processing	SARS-CoV-2 A372T S (closed) (PDB-7FCE EMDB-31525)	SARS-CoV-2 A372T S (open) (PDB- 7FCD EMDB-31524)
Magnification	×64000	×64000
Voltage (kV)	300	300
Electron exposure (e–/Å^2)	50	50
Defocus range (μm)	-1.5 to -1.8	-1.5 to -1.8
Pixel size (Å)	1.0979	1.0979
Symmetry imposed	C1	C1
Initial particle images (no.)	928,347	928,347
Final particle images (no.)	348,420	55,181
Map resolution (Å)	3.1	3.9
FSC threshold	0.143	0.143
Map resolution range (Å)	3.1-5	3.9-6
**Refinement**		
Initial model used (PDB code)	6VXX	6VYB
Model resolution (Å)	3.1	3.9
FSC threshold	0.143	0.143
Model resolution range (Å)	3.1	3.9
Map sharpening B factor (Å^2)	-114.8	-133.542
**Model composition**		
Non-hydrogen atoms	25671	24488
Protein residues	3125	3057
Ligands	84	37
**B factors (Å^2)**		
Protein	99.53	205.09
Ligand	122.05	30.00
**R.m.s. deviations**		
Bond lengths (Å)	0.010	0.013
Bond angles (°)	1.207	1.464
**Validation**		
MolProbity score	1.28	2.28
Clashscore	1.88	10.46
Poor rotamers (%)	0.88	1.94
**Ramachandran plot**		

Favored (%)	95.28	91.77
Allowed (%)	4.56	7.96
Disallowed (%)	0.16	0.27
**Privateer**		
Wrong anomer	0	0
Stereochemical problems	0	0
Unphysical puckering amplitude	0	0
In unlikely ring conformation	0	0
